# Pediatric Liver Disease Patients and Secondary Glycosylation Abnormalities

**DOI:** 10.3389/fped.2020.613224

**Published:** 2021-01-13

**Authors:** Anna Bogdańska, Patryk Lipiński, Paulina Szymańska-Rożek, Irena Jankowska, Piotr Socha, Anna Tylki-Szymańska

**Affiliations:** ^1^Department of Biochemistry, Radioimmunology and Experimental Medicine, The Children's Memorial Health Institute, Warsaw, Poland; ^2^Department of Paediatrics, Nutrition and Metabolic Diseases, The Children's Memorial Health Institute, Warsaw, Poland; ^3^Faculty of Mathematics, Informatics and Mechanics, University of Warsaw, Warsaw, Poland; ^4^Department of Gastroenterology, Hepatology, Feeding Difficulties and Pediatrics, The Children's Memorial Health Institute, Warsaw, Poland

**Keywords:** congenital disorder of glycosylation (CDG), acute liver failure (ALF), acute liver injury (ALI), chronic liver disease (CLD), liver transplantation, serum transferrin isoelectrofocusing, autoimmune hepatitis (AIH)

## Abstract

**Background:** Isoelectric focusing (IEF) of serum transferrin (Tf) is still the method of choice for diagnosis of congenital disorders of glycosylation (CDG). An abnormal glycosylation is also a known phenomenon in adult liver disease patients. The aim of this study was to characterize glycosylation disturbances in pediatric patients with primary liver disease. However, there are no reports of this phenomenon in children.

**Materials and Methods:** Between 1995 and 2019, circa 2,000 serum Tf isoform analyses have been performed in children with primary liver diseases; some of them underwent subsequent analyses. We enrolled in this study 19 patients who developed an acute liver injury (ALI)/failure (ALF) or exhibited a chronic liver disease (CLD) and were evaluated and listed for liver transplantation (LTx) or had just undergone this procedure, and secondary abnormal serum Tf isoform profile.

**Results:** Among 12 patients with ALI/ALF, 10 had an increased percentage of asialo-, monosialo-, and disialo-Tf isoforms. All patients with CLD had an increased percentage of asialo- and monosialo-Tf isoform. Two patients diagnosed with recurrent ALF had very specific serum Tf profile with a huge increase in the asialo- and monosialo-Tf isoform. On follow-up analyses (available in some patients), serum Tf IEF profile normalized in parallel to normalization of liver function tests, spontaneously or during treatment, including glucocorticosteroids in AIH, LTx in CLD.

**Conclusions:** All pediatric patients with primary liver disease had increased asialo-Tf as well as monosialo-Tf isoforms. None of them had elevated percentage of trisialo-Tf isoform.

## Introduction

Liver is the main site of synthesis and physiological activity of transferrin (Tf) and other serum glycoproteins (1). Isoelectric focusing (IEF) of serum Tf is still the method of choice for diagnosis of congenital disorders of glycosylation (CDG), especially N-glycosylation disorders associated with sialic acid deficiency (2, 3). Transferrin is the iron-binding serum glycoprotein which is produced in hepatocytes and contains two N-linked glycosylation sites (4, 5). An abnormal glycosylation is also a known phenomenon in liver disease patients; it has been described in alcoholic liver disease (decreased enzyme activities of mannosyltransferase and galactosyltransferase, lowered intracellular dolichol concentration, desialylation of serum Tf, α1-antitrypsin and ceruloplasmin; hyperfucosylation of haptoglobin and serum Tf), galactosemia and fructosemia (the pattern resembling CDG type I), chronic hepatitis B and C, non-alcoholic steatohepatitis, and bile-related liver diseases (6–21). Despite of IEF, there are also some other methods, like HPLC or MALDI-TOF but they are not available in Poland. Specifically, carbohydrate deficient transferrin (CDT) analysed using isoelectric focusing (IEF) as a sum of asialo-, monosialo-, and disialo-Tf isoforms has been widely used in recent years as a marker of chronic alcohol abuse in adults. However, the literature about serum Tf isoform profiles in children with liver disease is sparse. The aim of this study was to characterize glycosylation disturbances in pediatric patients with primary liver disease.

## Materials and Methods

Between 1995 and 2019 years, about 2,000 serum Tf isoform analyses have been performed in children with primary liver diseases hospitalized in our Institute. Some of the patients underwent subsequent analyses during follow-up. Serum Tf isoforms were analyzed by isoelectrofocusing (IEF) agarose gel electrophoresis according to the method described by Van Eijk et al. (5).

**Figure 1:**
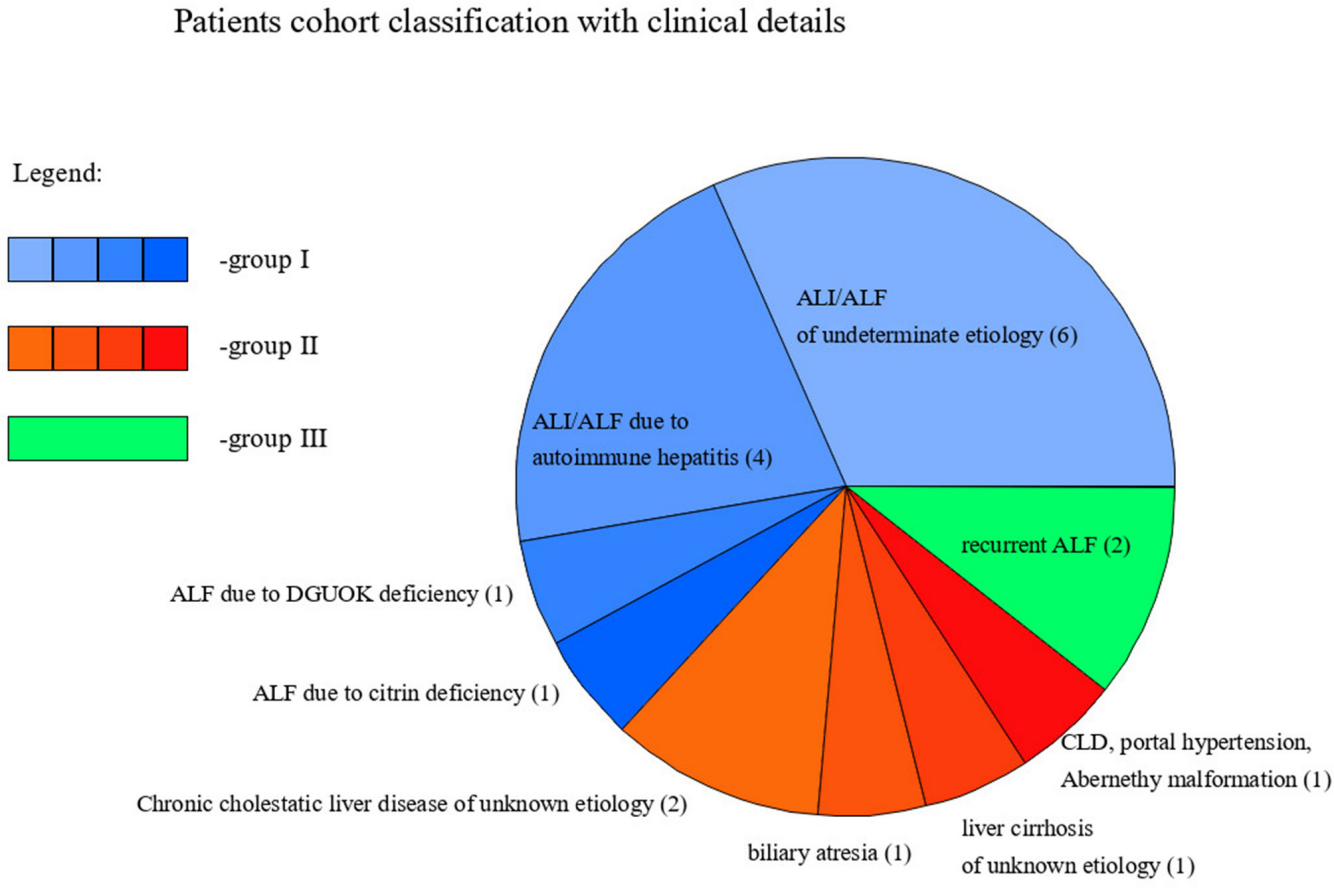
Patients cohort classification with clinical details.

Serum Tf consists of the mixture of isoforms and has two iron binding sites, which was saturated with iron (20 μl serum with 80 μl 0,9% NaCl, 2 μl 10 mM Fe(III) citrate and 2 μl 0.1 M NaHCO_3_). Transferrin migrated through a 1% agarose gel (45 mg of agarose were added to 4.5 ml of distilled water) with 5% ampholines in a pH range of 5.0-7.0 on a Multiphore 2117 apparatus (LKB) with a modified electrode lid. Tf isoforms were visualized by immunofixation (200 μl polyclonal rabbit anti-human transferrin serum) and 0.5% Coomasie Brillant Blue solution staining, as presented in Figure 1. The percentage of Tf fractions was assessed densitometrically and carbohydrate deficient transferrin value (% CDT) was measured as a sum of asialo-, monosialo-, and disialo-Tf isoforms.

We enrolled in this study 19 patients with primary liver disease, who developed an acute liver injury/failure or exhibited a chronic liver disease, and secondary abnormal serum Tf isoform profile. They are the totality of patients who showed glycosylation abnormalities among patients with primary liver disease. Patients presenting an abnormal serum Tf isoform profile secondary to galactosemia (galactose-1-phosphate uridyltransferase deficiency) or fructosemia (fructose 1-phosphate aldolase deficiency) were excluded from the study.

Acute liver injury (ALI) was characterized by an acute hepatocellular damage or death, expressed by elevation of serum transaminases, leading to rapid loss of hepatocellular function, excretory (cholestasis defined as serum conjugated bilirubin > 1,0 mg/dL according to the latest ESPGHAN recommendations) and synthetic (coagulopathy defined as INR > 1.2), in a patient without chronic liver disease (22–24). Pediatric acute liver failure (ALF) was diagnosed based on the above-mentioned criteria and one of the following: hepatic-based coagulopathy defined as INR ≥ 1.5 not corrected by vitamin K in the presence of clinical hepatic encephalopathy, or INR ≥ 2.0 regardless of the presence or absence of clinical hepatic encephalopathy (22, 23). The term ‘recurrent ALF' (RALF) was used to describe patients with repeated episodes of acute liver injury with recovery of hepatic function between crises (25, 26).

All the patients with ALI/ALF were evaluated according to the current clinical practical guidelines (23). In some of them, liver biopsy was essential to make a diagnosis, i.e., autoimmune hepatitis (AIH). There were also patients in whom an undetermined etiology of ALI/ALF was established.

Chronic liver disease (CLD) refers to liver disease that lasted over a period of at least 6 months. In this group were enrolled patients evaluated and listed for liver transplantation (LTx) or who had just undergone this procedure (27).

The retrospective chart review of patients' medical long-term records concerning the demographics, clinical characteristics, and biochemical data, including serum Tf IEF profile, aspartate (AST) and alanine (ALT) aminotransferases, international normalized ratio (INR), total and direct serum bilirubin, gamma-glutamyl transpeptidase (GGT) were collected. Ethical approval was obtained from the Children's Memorial Health Institute Bioethical Committee, num 23/KBE/2020, Warsaw, Poland.

Statistical analysis was conducted in R software and consisted of: first, calculating the mean of the observed levels of Tf isoforms and liver tests results in each patients (some of them had only one measurement, but some had even six measurements taken). Next, simple *t*-tests or Welsch *t*-tests (if the variances differed between patients groups) were performed in order to check if the three groups are different in terms of the measured level of Tf isoforms or liver test results. Since for every parameter we compare group I with group II, group II with group III, and group I with group III, we account for multiple comparisons by p-value adjustments using Bonferroni–Holm method (28). Finally, within every group, we check if there is a correlation between Tf isoforms % and liver tests results by calculating Pearson's linear correlation coefficient r. We also check if the obtained coefficients are statistically significant, accounting for multiple comparisons using Benjamini–Hochberg procedure (29).

## Results

### Clinical and Biochemical Results

Nineteen pediatric patients (13 males and 6 females) with primary liver disease presented secondary N-hypoglycosylation of serum Tf. The mean age at diagnosis was 3 years and 1 month. Based on the phenotype of liver disease, patients were divided into 3 groups, as following: group I—acute presentation, including ALI/ALF; group II—chronic presentation, including CLD; group III—recurrent ALF (RALF).

Detailed characteristics of clinical and biochemical features are summarized in Table 1.

**Table 1:** Detailed characteristics of clinical and biochemical features of study patients.

**Number/Sex**	**Age**	**Asjalo-*N*: 0**	**Monosjalo- *N*: 0**	**Disjalo-*N*: 1,5–6,2**	**Trisjalo- *N*: 7,4–17,1**	**Tetrasjalo-*N*: 55,7–67,2**	**Pentasjalo- *N*: 13,2–19,9**	**Heksasjalo-*N*: 2,5–5,6**	**AST**	**ALT**	**INR**	**Total/direct serum bilirubin**	**GGT**	**Clinical outcome**
**Group I**														
1/F	11 m	1,4	2,5	11,1	13,2	46,9	18,6	6,3	1,615	349	0,92	8,9/7,0	820	ALI of undeterminate etiology
	12 m	4,9	4,1	6,3	13,7	45,8	19,1	6,0	1,081	425	0,92	5,0/4,6	675	
	14 m	0,5	2,2	6,6	12,9	49,4	20,9	7,5	333	252	0,85	2,85/2,57	481	
2/M	1 m	0,8	1,9	3,3	8,7	61,6	19,0	4,5	51	32	3,3	7,96/1,82	249	ALF due to DGUOK deficiency
	3 m	0,8	1,4	5,2	11,7	58,3	18,1	4,4	149	53	1,22	5,2/4,1	390	
	4 y	0	0	5,6	14,2	58,4	19,0	2.8	47	30	0,96	Normal	12	
3/M	7 y 4 m	0,4	1,2	7,4	12,4	50,3	21,6	6,7	2,277	1,247	1,45	21,5/19,9	107	ALI due to autoimmune hepatitis
	7 y 5 m	0,2	0,9	7,2	12,5	46,8	24,8	7,5	510	721	1,2	19,9/16,6	211	
	7 y 8 m	0	0	5,2	12,1	56,8	21,7	4,2	30	39	1,02	Normal	59	
4/M	2 y 7 m	0,9	1,6	6,4	11,2	50,1	23	6,8	1,475	1,173	1,32	8,4/7,3	198	ALI of undeterminate etiology
	2 y 8 m	1,0	2,0	7,3	12,5	46,2	21,4	6,9	185	155	1,05	15,1/12,3	97	
5/F	13 y 4 m	0,9	1,2	6,7	9,6	53	22,8	5,8	855	587	2,27	5,9/4,1	48	ALF due to autoimmune hepatitis
6/F	11 y 4 m	2,3	4,2	6,0	9,9	48,4	22,1	7,1	90	34	2,3	3,8/2,2	n.a.	ALF of undeterminate etiology
	11 y 7 m	1,5	3,2	7,3	12,6	52,8	17,8	4,8	71	79	1,98	7,1/5,3	60	
7/M	5 y 8 m	0,6	1,6	6,3	12,7	49,4	21,5	7,9	2,716	1,678	1,27	21,1/16,8	68	ALI due to autoimmune hepatitis
	6 y	0,6	1,1	7,2	13,6	46,3	23,1	8,0	2,094	1,494	1,23	21/20,2	75	
8/M	2 y 6 m	0,8	1,9	8,5	15,8	45,1	23,8	4,0	1,419	567	2,36	15,3/13,7	41	ALF of undeterminate etiology
9/M	11 m	0,9	1,1	6,2	17,6	46,6	22,4	5,1	2,040	1,470	1,49	26,1/19,5	n.a.	ALI of undeterminate etiology
10/F	15 m	2,4	1,9	5,8	7,8	54	22	5,1	1,082	264	1,69	6,2/5,2	90	ALI of undeterminate etiology
	16 m	N	N	N	N	N	N	N	24	20	1,05	Normal	27	
11/M	6 m	3,8	5,7	8,0	9,7	48	17,3	7,6	101	30	2,01	9,6/4,5	145	ALF due to citrin deficiency
12/F	7 y 6 m	0	0	6.5	11.2	47.9	27.8	6.6	2,213	1,784	1,46	15,9/13,3	122	ALI due to autoimmune hepatitis
	7 y 9 m	1,0	2,6	9,9	17,3	50,9	14,9	3,5	109	132	1,07	0.94/0,55	130	
	7 y 10 m	0	0	6,2	12,5	56,3	12,5	6,2	40	25	1,08	0,64/0,26	30	
**Group II**														
1/M	5 m	1,3	1,3	5,8	6,6	59,7	21,2	4,0	96	87	1,28	Normal	155	Liver cirrhosis of unknown etiology, LTx
	7 m (after LTx)	N	N	N	N	N	N	N	161	204	1,12	Normal	183	
	14 m (after LTx)	N	N	N	N	N	N	N	723	665	1,04	Normal	98	
2/M	10 m	0,8	1,5	2,9	9,6	58,5	21,5	5,2	157	95	1,33	16,6/12,8	41	Biliary atresia, listed for LTx
	11 m	1,0	1,3	5,2	11,7	57,3	19,7	3,7	174	88	1,21	17,5/14,1	72	
3/F	11 m	1,6	2,4	6,5	10,3	45,6	25,9	7,8	418	229	1,73	18,8/15,5	85	Chronic cholestatic liver disease of unknown etiology, LTx
4/M	7 m	1,8	1,3	5,1	10,9	48,4	24,3	8,2	329	174	1,43	15,7/13,9	185	Chronic cholestatic liver disease of unknown etiology, LTx
	7 y (after LTx)	0	0	4,9	13,4	59,9	19,1	2,8	36	42	0,98	Normal	28	
5/M	9 m	3,1	2,7	7,5	10,9	48,3	21,2	6,3	89	41	1,36	Normal	11	CLD, portal hypertension, Abernethy malformation, listed for LTx
	14 m	1,1	1,4	4,9	11,5	60,8	18,3	2,0	91	48	1,47	Normal	n.a.	
	16 m	0,2	0,3	3,8	11,9	59,2	22,1	2,6	103	38	1,38	Normal	n.a.	
**Group III**														
1/M	9 m	3,0	1,4	7,3	12,5	48,1	22,4	5,3	1,806	550	1,43	8,1/5,7	98	RALF (4 episodes)
	1 y 10 m	18,3	5,1	6,5	11,6	41,6	13,5	3,3	1,520	680	1,53	8,3/6,1	41	
	4 y 3 m	13,0	4,3	5,5	14,6	49,7	10,0	2,5	2,280	860	2,1	9,3/7,1	68	
	5 y 3 m	0	0	5,2	11,5	57	21,3	5,0	26	26	0.9	Normal	30	
	7 y 3 m	14,2	2,2	3,3	5,9	47,5	19,9	7,1	1,240	450	2,1	3,9/3,5	90	
	8 y	0	0	2,7	5,9	67,5	19,5	4,2	20	22	0.9	Normal	15	
2/M	5 m	6,9	2,8	5,9	10,9	42,9	22,6	8,7	1,350	580	1,67	8,2/6,9	60	RALF (4 episodes)
	3 y 2 m	20,9	2,4	3,6	7,1	47,4	15,6	2,9	1,270	385	1,9	10,5/9,4	65	
	4 y 10 m	0	0	2,4	9,2	65,3	19,2	3,5	28	26	0.95	Normal	30	
	5 y	20,6	4,6	5,3	9,0	44,9	12,9	2,6	1,650	580	1,5	8,6/8,2	50	
	5 y 2 m	0	0	3,2	9,8	51,8	28,9	6,4	30	20	0,9	Normal	26	
	9 y	17,9	5,7	5,2	8,9	46,9	12,5	3,2	1,200	420	1,4	8,0/7,2	45	
	4 y 7 m	0	0	6,3	14,8	48,1	25,9	7,8	315	143	1,03	0,81/0,48	73	
	4 y 9 m	0	0	6,3	14,2	48,6	25,5	5,4	1,128	496	1,05	0,92/0,57	489	

Out of 19 patients, 14 presented ALI/ALF, including 4 with AIH, 1 with citrin deficiency, 1 with deoxyguanosine kinase (DGOUK) deficiency, 6 with an indeterminate etiology, and 2 with recurrent ALF (RALF). The mean age at diagnosis was 4.5 years.

In the group of 5 patients with CLD, 1 had portal hypertension due to Abernethy malformation and was listed for LTx; 1 had biliary atresia and was listed for LTx; 2 had chronic cholestatic liver disease of an unknown etiology and had undergone LTx; one was diagnosed with liver cirrhosis of unknown etiology and had undergone LTx. The mean age at diagnosis was 8 months.

We present the classification of patients with these clinical details in Figure 1.

Among 12 patients from group I, 10 had an increased percentage of asialo-, monosialo-, and disialo-Tf isofroms. Follow-up analyses were available only in four patients (including two with AIH); serum Tf IEF profile normalized in parallel to the normalization of liver function tests (in patients with AIH on treatment with glucocorticosteroids).

Among five patients from group II, all had an increased percentage of asialo- and monosialo-Tf isoforms while two of them had also increased disialo-Tf isoform. Follow-up analyses were available only in two patients, after LTx; serum Tf IEF profile normalized in parallel to the normalization of liver function tests.

Group III included two patients diagnosed with RALF and an increased percentage of asialo- (huge increase) and monosialo-Tf isoforms, and in one of them also disialo-Tf isoform on some of the results. Between episodes of ALF, serum Tf isoform profile was normal as well as liver function tests.

Regarding particular serum Tf isoforms, all patients had an increase in asialo-Tf as well as monosialo-Tf isoform. An elevated percentage of disialo-Tf isoform was observed in 12 out of 14 patients with an acute hepatic injury, including 10 out of 12 from group I and 2 from group III (transient elevation). None of the patients had elevated percentage of trisialo-Tf isoform.

We include averages of % Tf isoforms, as well as liver test results for each group, along with reference ranges (for Tf isoforms) in Table 2.

Table 2Averages of Tf % isoforms **(A)** and liver function test **(B)** for the three groups of patients.

**Table:** 

**(A)**	**Asiao-**	**Monosialo-**	**Disialo-**	**Trisialo-**	**Tetrasialo-**	**Pentasialo-**	**Heksasialo-**
Group I	1.2	1.92	6.72	12.2	50.6	21.1	5.84
Group II	1.23	1.44	5.35	10.2	54.7	22	5.08
Group III	9.67	2.38	4.68	9.74	50.9	18.2	4.56

**Table:** 

**(B)**	**AST**	**ALT**	**INR**	**Total bilirubin**	**GGT**
Group I	952	6,156	1.66	11	111
Group II	237	158	1.35	13.1	80.9
Group III	1,035	383	1.44	8.11	51.5

### Data Visualization and Statistical Analysis

**Figure 2:**
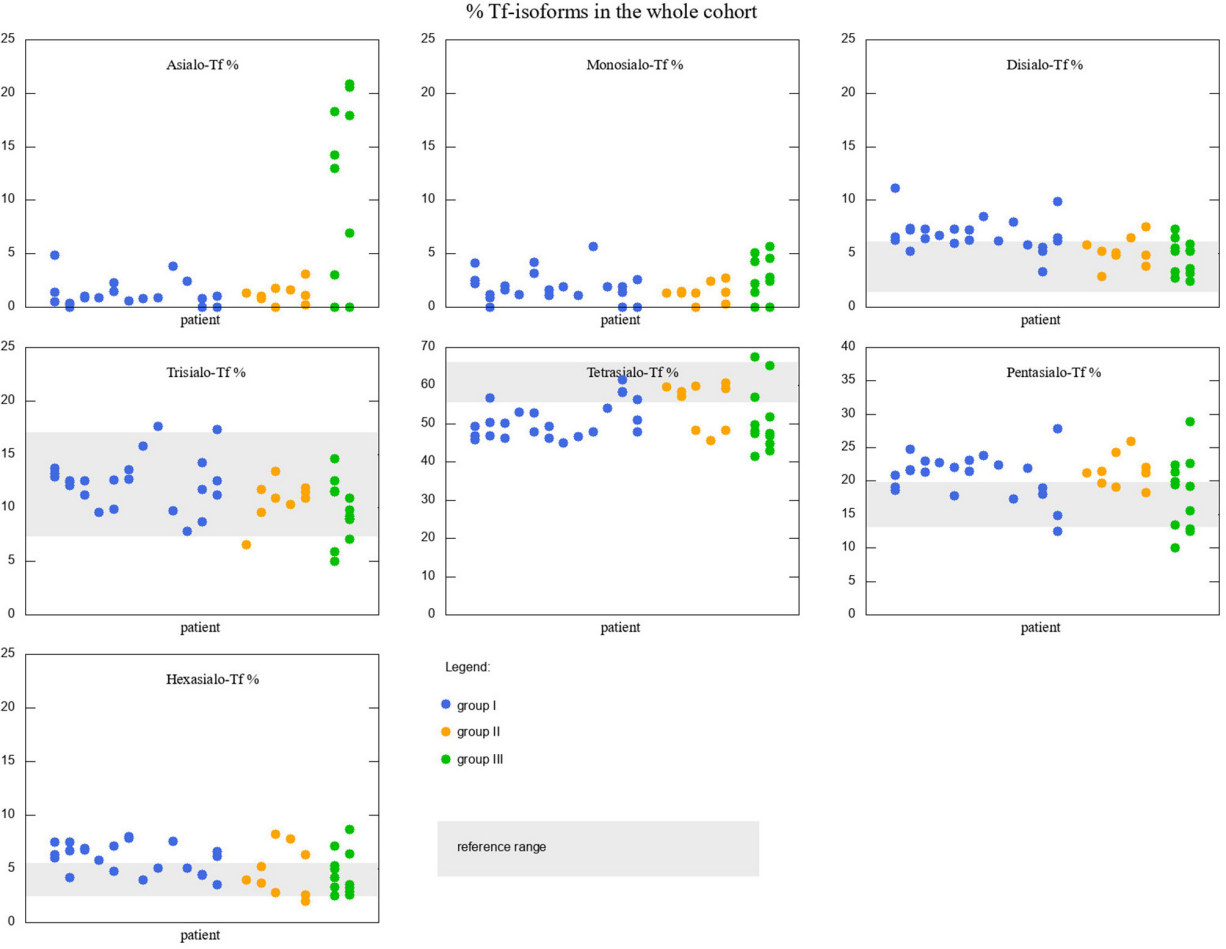
Percentage of Tf-isoforms in the whole cohort of studied patients. Reference ranges marked with a gray rectangle (reference range for Asialo-Tf and Monosialo-Tf is 0). Groups I, II, and III marked with different colors. Note the change in y-axis range for Tetrasialo-Tf and Pentasialo-Tf.

Serum Tf isoform results for the whole cohort of patients are depicted in Figure 2. Different colors are used to distinguish between the three different groups of patients. Reference ranges are visualized with gray rectangles. Some of the patients had more than one measurement performed, therefore in some places there are several values associated to one argument. Although the picture might give an idea about how Tf isoforms vary between the groups, we performed simple *t*-tests or Welsch *t*-test to check if the differences in means are statistically significant. We found that:

- Group I is different from group II in disialo-Tf (mean in group I = 6.72%, mean in group II = 5.35%; *p*-value = 0.02 ≈ α/3),
- Group II differs from group III in asialo-Tf (mean in group II = 1.23%, mean in group III = 9.57%; *p*-value = 0.00016 < α/3), and pentasialo-TF (mean in group II = 22%, mean in group III = 18.2%; *p*-value = 0.01789 < α/2),
- Group I is different from group III in pentasialo-Tf (mean in group II = 21.1%, mean in group III = 18.2%; *p*-value = 0.00016 < α/3)

**Figure 3:**
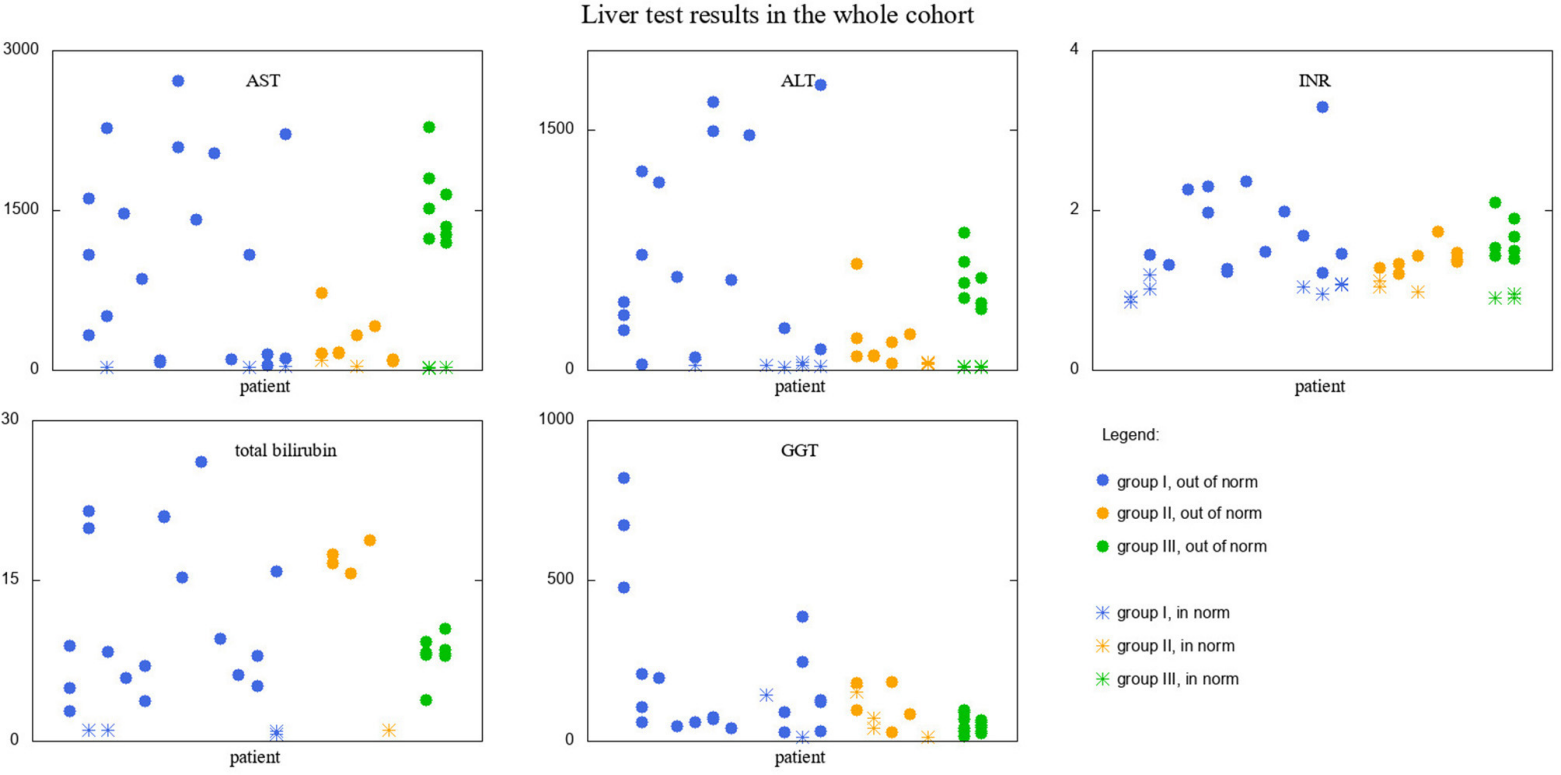
Liver test results in the whole cohort of studied patients. Results falling into respective norms (for the given parameter and age) marked with a different point type than those falling outside the norm. Groups I, II, and III marked with different colors.

Similar analysis was performed for liver function tests results. The visualization of data is presented in Figure 3. Since the reference ranges are different for different age ranges, the results falling into norms are depicted with different markers than those falling outside. *T*-test results revealed that:

- Group I is significantly different from group III in GGT results (111.2 and 51.5, *p*-value = 0.011 < α/3).

We also investigated whether there are correlations between Tf-isoform profiles and liver function test results within the groups. Interestingly, we found no correlations in group I at all. In group II, we found several correlations (Supplementary Table 1), but only two of them were statistically significant: between INR results and monosialo-Tf, and INR and tetrasialo-Tf. In group III, since there are only two patients, and we averaged the results, we obtained only two data points for every pair Tf-isoform %—liver parameter, which is insufficient for sensibly investigating correlations.

**Figure 4:**
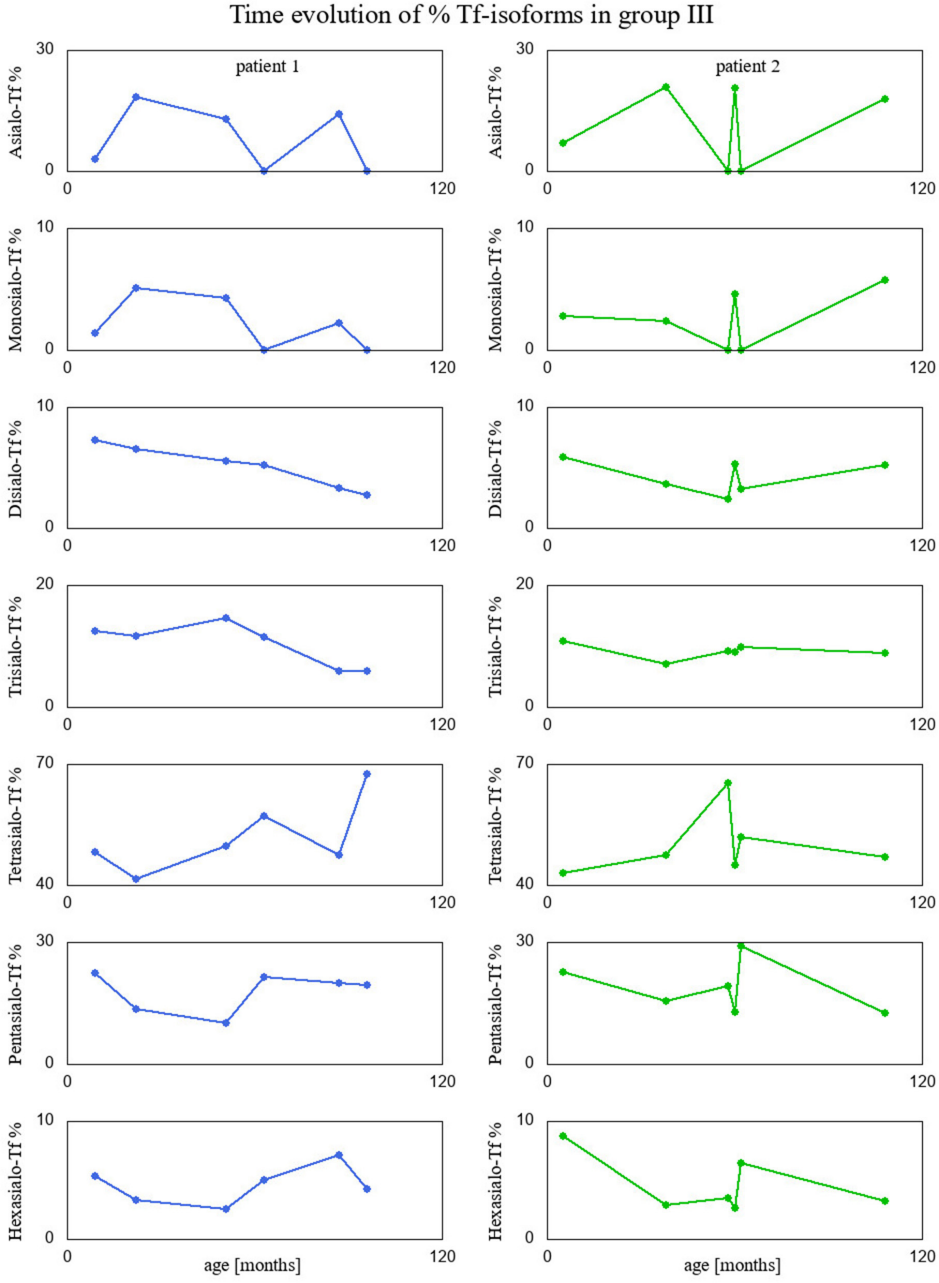
Percentage of Tf-isoforms with respect to age (in months) for the patients from group III.

**Figure 5:**
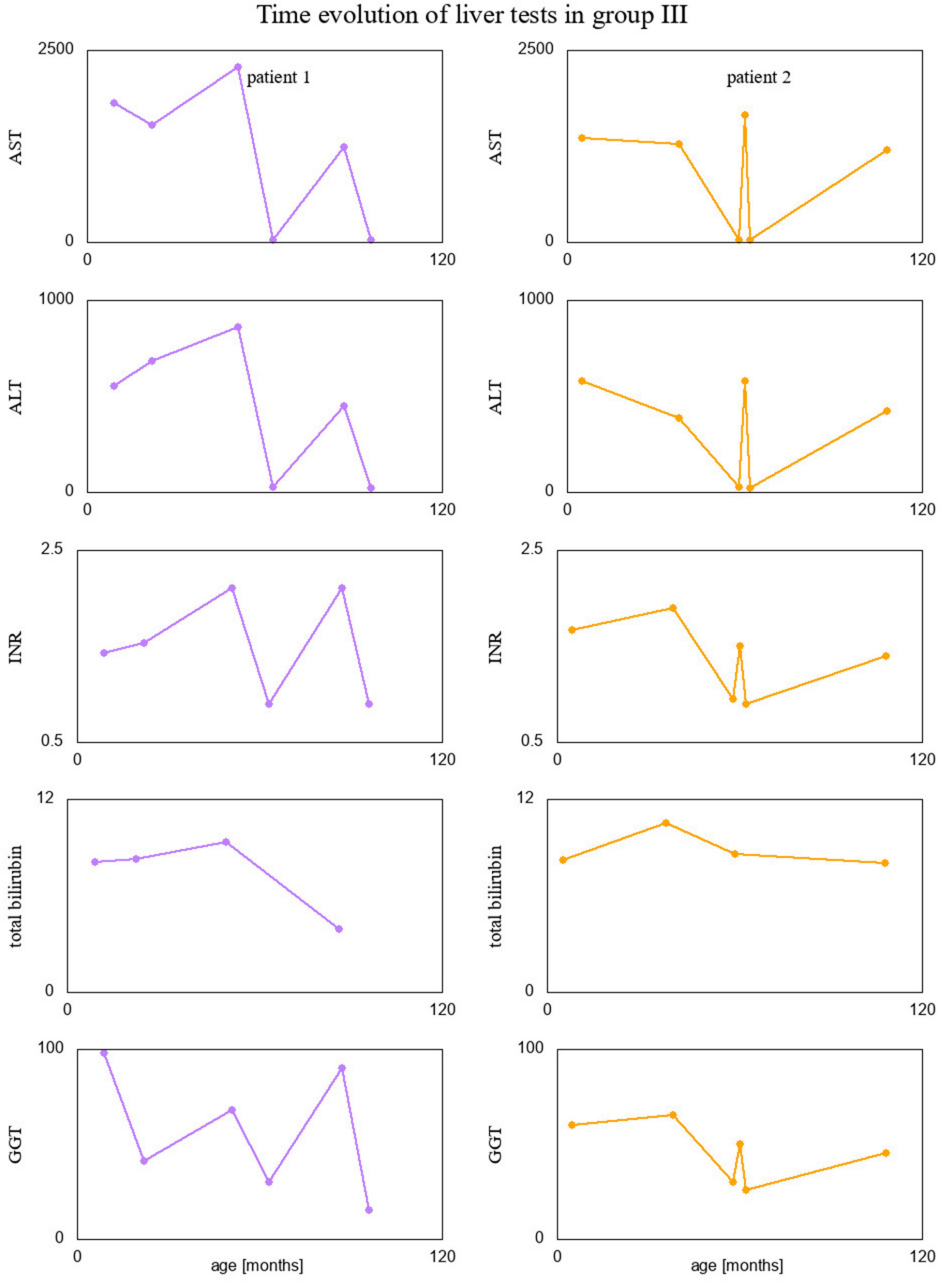
Liver test results with respect to age (in months) for the patients from group III.

Finally, we visualized time evolution of serum Tf-isoform % and liver function test results in patients form group III, since they had several measurements taken over time. These interesting visualizations are given in Figures 4, 5.

## Discussion

To the best of our knowledge, this is the first study aiming to characterize pediatric liver disease patients with secondary serum Tf N-hypoglycosylation.

Fourteen out of 19 patients in the study had an acute presentation of liver disease in the form of ALI/ALF or recurrent ALF. Secondary N-hypoglycosylation of serum Tf as a mild increase in the asialo-, monosialo-, and disialo-Tf isoforms was diagnosed. However, we observed no correlation between serum Tf isoforms and liver function tests globally, i.e., in the whole cohort of patients. On the other hand, we found correlations of serum Tf isoforms and liver function tests in group II. On follow-up analyses, serum Tf IEF profile normalized in parallel to normalization of liver function tests, spontaneously or during treatment, including glucocorticosteroids in AIH. The mechanism of secondary serum Tf N-hypoglycosylation in the study group was a severe liver impairment, the exact incidence of this phenomenon is not elucidated. Due to its half-life of approximately 2 weeks, there is a lag between normalization of liver enzymes and improvement of transferrin glycosylation. This fact rules out the usage of serum transferrin glycosylation as a (secondary) marker for ALI/ALF in the absence of previous measurements.

Interestingly, two patients presenting recurrent acute liver failure (RALF) had very specific serum Tf IEF profile with a huge increase of asialo-Tf isoform during episodes of ALF. The mechanism of those abnormalities reflects a severe liver impairment and secondary Tf N-hypoglycosylation with a decrease of tetrasialo-Tf and an increase in asialo-Tf.

Between episodes of ALF, liver function recovered completely together with serum Tf IEF profile. This is the first report on secondary serum Tf N-hypoglycosylation in the course of RALF. Pathogenic variants in *NBAS, RINT1*, or *LARS* genes have been identified as a cause of RALF (25, 26). Whole exome sequencing (WES) analysis did not identify the genetic background in our patients.

Besides patients with ALI/ALF, our study group included five patients with serum Tf N-hypoglycosylation secondary to chronic liver disease CLD; three of them underwent LTx. Sixty percent of patients were diagnosed with chronic cholestatic liver disease; the exact mechanism of serum Tf N-hypoglycosylation remains unknown but it could be related to an impaired excretory (and sometimes also synthetic) hepatocellular function. A chronic liver disease impairs the serum Tf glycosylation, thus a decrease of tetrasialo-Tf is observed. Cellular mechanisms of liver regeneration in the course of chronic disease need to be also taken into account.

During follow-up after LTx, the serum Tf IEF profile normalized. This is the first report on serum Tf N-hypoglycosylation in children secondary to chronic cholestatic liver disease and its normalization after LTx.

A recently published paper by Jansen et al. about secondary glycosylation defects in adult patients with liver disease comprises the biggest cohort in the literature, reported up to now (30). Out of 961 enrolled patients, including those qualified for LTx or with chronic liver disease, 247 patients (26%) had hyposialylation of serum Tf. The authors did not identify a typical CDG type 1 pattern, while the majority of patients (70%) had an increase in the trisialo-Tf isoform. Regarding other patients, 42 (17%) had an elevated percentage of monosialo-Tf isoform and 4 (2%) had an elevated percentage of disialo-Tf isoform. None of the patients had an increase of all isoforms, a feature of most type II CDG.

**Figure 6:**
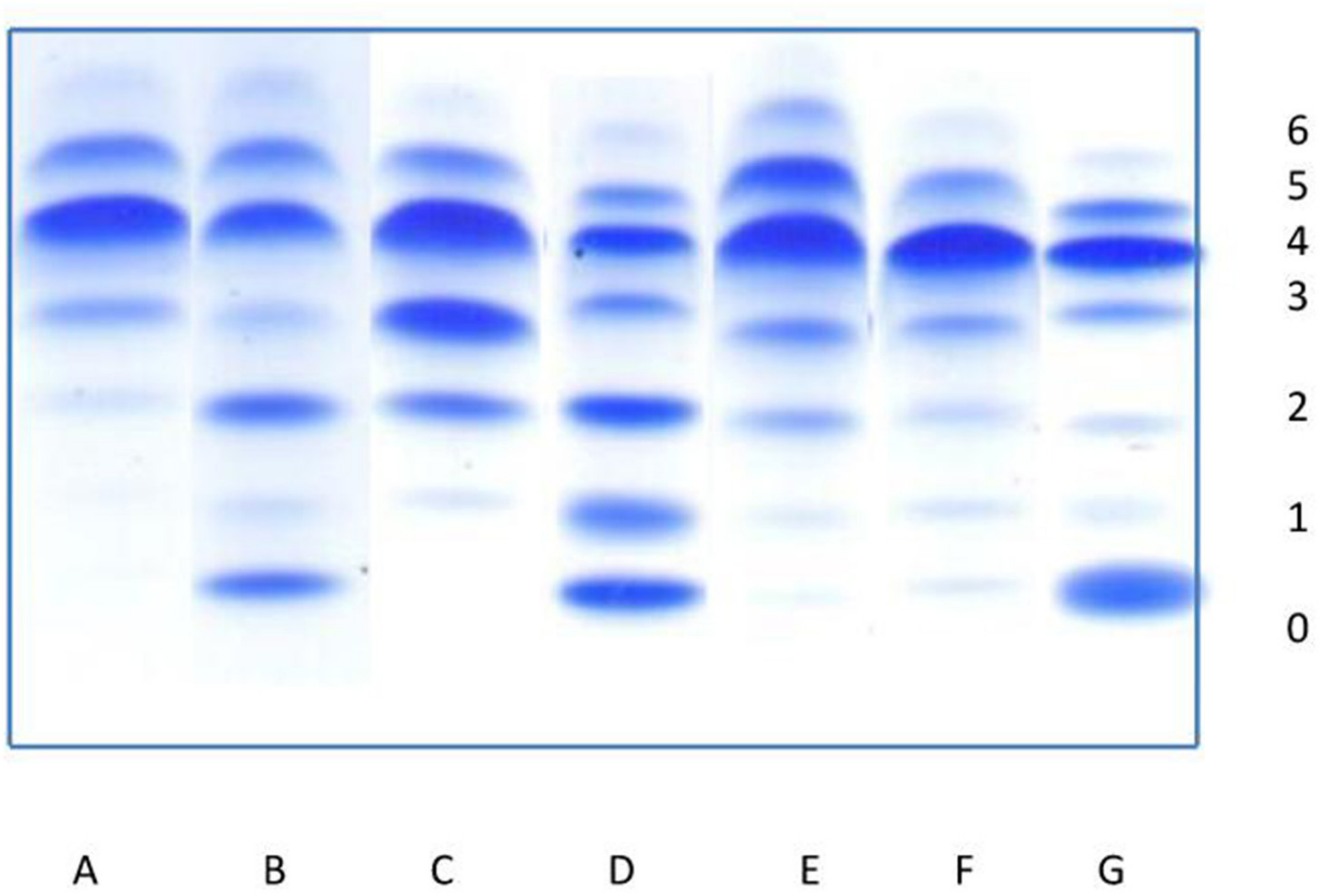
Isoelectrofocusing (IEF) of serum transferrin isoforms (0-asialotransferrin, 1-monosialotransferin, 2-disialotransferrin, 3-trisialotransferrin, 4-tetrasialotransferrin, 5-pentasialotransferrin, 6-hexasialotransferrin). **(A)** Normal profile. **(B–D)** Congenital disorders of glycosylation: **(B)** pattern for patient with CDG type I, **(C)** pattern for patient with CDG type II, **(D)** pattern for patient with mixed CDG I/II. **(E–G)** Secondary disorders of glycosylation: **(E)** slightly elevated asialo-, monosialo-, and disialotransferrin (group I), **(F)** slightly elevated asialo- and monosialotransferrin (group II), **(G)** highly elevated asialotransferrin, slightly monosialotransferrin (group III).

The dominant isoform in healthy individuals is tetrasialo-Tf (55.7–67.2%), while asialo- and monosialo-Tf isoforms are usually not detectable (4, 5). In CDG-I, there is an increase in asialo- and disialo-Tf, and a decrease in tetrasialo-Tf, whereas in CDG-II there is an increase in asialo-, monosialo, disialo-, and trisialo-Tf (2, 3). Figure 6 presents the exemplary Tf IEF isoform profiles for each study group in comparison to patients with CDG type I, type II, and type I/II.

An increase in the asialo-, monosialo-, and disialo-Tf isoforms could be related to endoplasmic reticulum (ER) dysregulation secondary to hepatocytes damage (1, 3). ER stress has been observed in a variety of liver diseases and since hepatocytes are enriched in both smooth and rough ER due to its metabolic functions, serum Tf isoforms could be used as a potential biomarker in pediatric liver disease (31–33). However, further studies are needed to elucidate the serum Tf isoforms in pediatric liver disease patients.

Since more convenient methods such as high performance liquid chromatography (HPLC) and ESI-Q-TOF or MALDI-TOF are available in selected laboratories, a further analytical strategy to characterize the abnormal serum Tf profile is needed.

## Conclusions

All pediatric patients with primary liver disease had an increase in asialo-Tf as well as monosialo-Tf isoform. None of them had an elevated percentage of trisialo-Tf isoform.

This is the first report on secondary serum Tf N-hypoglycosylation in the course of RALF.

On follow-up analyses, serum Tf IEF profile normalized in parallel to normalization of liver function tests, spontaneously, or during treatment, including glucocorticosteroids in AIH.

The exact mechanism of serum Tf N-hypoglycosylation in pediatric liver disease patients remains unknown but it could be related to a severe hepatocellular impairment.

The only liver function test result that could be useful for distinguishing between the groups of patients is GGT—it is much lower for group III than for group I.

## Data Availability Statement

The original contributions presented in the study are included in the article/Supplementary Materials, further inquiries can be directed to the corresponding author/s.

## Ethics Statement

The studies involving human participants were reviewed and approved by Children's Memorial Health Institute Bioethical Committee, num 23/KBE/2020. Written informed consent to participate in this study was provided by the participants' legal guardian/next of kin.

## Author Contributions

AT-S, PL, and AB: project administration. AT-S: supervision. PL, AB, IJ, PS, and AT-S: investigation. PS-R: statistical analysis. PL and AB: writing—original draft. PL, AB, PS-R, and AT-S: writing—review and editing. All authors contributed to the article and approved the submitted version.

## Conflict of Interest

The authors declare that the research was conducted in the absence of any commercial or financial relationships that could be construed as a potential conflict of interest.

## Supplementary Material

Supplementary Table 1 Pearson's correlation coefficients between % isoforms and liver function test for group II. Correlation coefficient values, for which the *p*-value ≤ 0.05 marked with color (reddish for positive, bluish for negative).
