# Supplementary material for: Pediatric Liver Disease Patients and Secondary Glycosylation Abnormalities
Source: Front Pediatr. 2021 Jan 13;8:613224. doi: 10.3389/fped.2020.613224 (PMC7838542; doi:10.3389/fped.2020.613224)
Supplement: Supplementary Table 1 — Pearson's correlation coefficients between % isoforms and liver function test for group II. Correlation coefficient values, for which the p-value ≤ 0.05 marked with color (reddish for positive, bluish for negative). [file Table_1.DOC]

| B | Asialo- | Monosialo- | Disialo- | Trisialo- | Tetrasialo- | Pentasialo- | Hexasialo- |
| --- | --- | --- | --- | --- | --- | --- | --- |
| AST | 0.47786 | 0.617136 | 0.734878 | -0.54719 | -0.55343 | 0.814691 | 0.707673 |
| ALT | 0.333254 | 0.299261 | 0.609932 | -0.86505 (p = 0.058) | -0.07659 | 0.420736 | 0.274184 |
| INR | 0.711676 | 0.880604 (p = 0.048) | 0.592089 | 0.258623 | -0.89767 (p = 0.039) | 0.827448 | 0.748328 |
| Total bilirubin | -0.32081 | 0.166412 | -0.00313 | -0.37627 | -0.42912 | 0.540075 | 0.711573 |
| GGT | -0.16673 | -0.20611 | 0.285542 | -0.63869 | 0.08517 | 0.205566 | 0.202261 |
